# Supplementary material for: Sphingosine 1-phosphate-regulated transcriptomes in heterogenous arterial and lymphatic endothelium of the aorta
Source: eLife. 2020 Feb 24;9:e52690. doi: 10.7554/eLife.52690 (PMC7054001; doi:10.7554/eLife.52690)
Supplement: Transparent reporting form [file elife-52690-transrepform.docx]

***eLife’s* transparent reporting form**

We encourage authors to provide detailed information *within their submission* to facilitate the interpretation and replication of experiments. Authors can upload supporting documentation to indicate the use of appropriate reporting guidelines for health-related research (see [EQUATOR Network](http://www.equator-network.org/%20)), life science research (see the [BioSharing Information Resource](https://biosharing.org/)), or the [ARRIVE guidelines](http://www.plosbiology.org/article/info:doi/10.1371/journal.pbio.1000412) for reporting work involving animal research. Where applicable, authors should refer to any relevant reporting standards documents in this form.

If you have any questions, please consult our Journal Policies and/or contact us: [editorial@elifesciences.org](mailto:editorial@elifesciences.org).

**Sample-size estimation**

- You should state whether an appropriate sample size was computed when the study was being designed
- You should state the statistical method of sample size computation and any required assumptions
- If no explicit power analysis was used, you should describe how you decided what sample (replicate) size (number) to use

Please outline where this information can be found within the submission (e.g., sections or figure legends), or explain why this information doesn’t apply to your submission:

For bulk RNA-seq and ATAC-seq experiments, three biological replicates are commonly used for statistical analyses, as discussed in Schurch et al., *RNA*, 2016 Jun;22(6):839-51 (PMID: 27022035).

**Replicates**

- You should report how often each experiment was performed
- You should include a definition of biological versus technical replication
- The data obtained should be provided and sufficient information should be provided to indicate the number of independent biological and/or technical replicates
- If you encountered any outliers, you should describe how these were handled
- Criteria for exclusion/inclusion of data should be clearly stated
- High-throughput sequence data should be uploaded before submission, with a private link for reviewers provided (these are available from both GEO and ArrayExpress)

Please outline where this information can be found within the submission (e.g., sections or figure legends), or explain why this information doesn’t apply to your submission:

- Bulk RNA-seq and ATAC-seq experiments were performed on three cohorts of mice, as described in the Figure Legends and Methods. Three cohorts of mice represent 3 biological replicates for bulk RNA-seq and ATAC-seq comparisons.
- scRNA-seq was performed on 1152 cells (768 GFP^high^ and 384 GFP^low^) sorted from 7 total female S1PR1-GS mice aged 8-12 weeks.
- All raw sequencing data have been submitted to GEO under accession ID GSE139065

**Statistical reporting**

- Statistical analysis methods should be described and justified
- Raw data should be presented in figures whenever informative to do so (typically when N per group is less than 10)
- For each experiment, you should identify the statistical tests used, exact values of N, definitions of center, methods of multiple test correction, and dispersion and precision measures (e.g., mean, median, SD, SEM, confidence intervals; and, for the major substantive results, a measure of effect size (e.g., Pearson's r, Cohen's d)
- Report exact p-values wherever possible alongside the summary statistics and 95% confidence intervals. These should be reported for all key questions and not only when the p-value is less than 0.05.

Please outline where this information can be found within the submission (e.g., sections or figure legends), or explain why this information doesn’t apply to your submission:

-Statistically significant transcripts and genomic intervals (i.e. peaks) from RNA-seq and ATAC-seq were determined using edgeR. The complete edgeR outputs from RNA-seq analyses are in Supplementary File 1, and the complete edgeR outputs from ATAC-seq analyses are in Supplementary File 3.

-In the manuscript, we provide graphical summaries of the edgeR outputs by showing “Volcano Plots”, which illustrate the p-value and fold-change associated with each transcript (RNA-seq) or peak (ATAC-seq).

-Other statistical tests used are reported in Figure Legends where appropriate.

(For large datasets, or papers with a very large number of statistical tests, you may upload a single table file with tests, Ns, etc., with reference to sections in the manuscript.)

**Group allocation**

- Indicate how samples were allocated into experimental groups (in the case of clinical studies, please specify allocation to treatment method); if randomization was used, please also state if restricted randomization was applied
- Indicate if masking was used during group allocation, data collection and/or data analysis

Please outline where this information can be found within the submission (e.g., sections or figure legends), or explain why this information doesn’t apply to your submission:

- The Methods section describes experimental groups. Mice of similar ages and the same sex were compared for individual biological replicates for bulk-sequencing experiments.

**Additional data files (“source data”)**

- We encourage you to upload relevant additional data files, such as numerical data that are represented as a graph in a figure, or as a summary table
- Where provided, these should be in the most useful format, and they can be uploaded as “Source data” files linked to a main figure or table
- Include model definition files including the full list of parameters used
- Include code used for data analysis (e.g., R, MatLab)
- Avoid stating that data files are “available upon request”

Please indicate the figures or tables for which source data files have been provided:

- Supplementary excel files with complete datasets or complete analysis outputs are provided. Each file is referenced in the manuscript.
- Source Code files include scripts used to center ATAC-seq paired-end reads on Tn5 cut sites (Source Code File 1), STAR code used for mapping of scRNA-seq .fastq files (Source Code File 2), and Pagoda2 R code used for scRNA-seq analysis (Source Code File 3).
